# Trends in Youth Fatal Drug Overdose and Suicide Intentionality

**DOI:** 10.1192/j.eurpsy.2024.919

**Published:** 2024-08-27

**Authors:** Y. Kaminer

**Affiliations:** Psychiatry, University of Connecticut, Farmington, United States

## Abstract

**Introduction:**

Fatal youth overdose (FYO) in the US has been driven by fentanyl and polysubstances since 2016. Youth suicide have also been increaing since the year 2000. The manner of FYO may be accidental, intentional or undetermined, Psychoactive drug use including opioids has been known to increase suicidality in youth.

**Objectives:**

Examine and compare the rate of intentinal and accidental FYO as well as specific drug toxicology in youth under 26 years of age in the state of Connecticut, USA; between the years 2016-2018 (Kaminer et al. JCASA 2020;29 80-87) and 2019-2021.

**Methods:**

We reviewed N=286 consecutive FYO case files of youth who died between 2019-2021, from the Connecticut office of the Chief Medical Examiner.

**Results:**

Comparing the periods of 2019-2021 2016-2018: A) FYO attributed to fentanyl increased significantly; B) Intentional YFO rates doubled from 3.8% to 7.7%; C) No gender differences were found between and within age groups; and D) hispanic rates increased significantly while caucasian rates decreased signficantly; F) for the first time YFO of youth under the age of 15 years was recorded and G) the age group of 15-19 years old constitute 10% of the YFO and remined unchanged.

**Conclusions:**

The use of lethal drugs leading to youth accidental and intentional FYO should be addressed by developing prevention-intervention approach. Focus on acute modifiable high-risk is prudent. The increase of intentional (i.e., suicidal) determined YFO is a major public health concern.

**Disclosure of Interest:**

None Declared

